# LGFUNet: A Water Extraction Network in SAR Images Based on Multiscale Local Features with Global Information

**DOI:** 10.3390/s25123814

**Published:** 2025-06-18

**Authors:** Xiaowei Bai, Yonghong Zhang, Jujie Wei

**Affiliations:** 1 Chinese Academy of Surveying and Mapping, Beijing 100036, China; 1291454630b@gmail.com (X.B.);; 2 Key Laboratory of Natural Resources Monitoring and Supervision in Southern Hilly Region, Ministry of Natural Resources, Changsha 410118, Hunan, China

**Keywords:** water extraction, SAR, deep learning, local feature, global information

## Abstract

To address existing issues in water extraction from SAR images based on deep learning, such as confusion between mountain shadows and water bodies and difficulty in extracting complex boundary details for continuous water bodies, the LGFUNet model is proposed. The LGFUNet model consists of three parts: the encoder–decoder, the DECASPP module, and the LGFF module. In the encoder–decoder, the Swin-Transformer module is used instead of convolution kernels for feature extraction, enhancing the learning of global information and improving the model’s ability to capture the spatial features of continuous water bodies. The DECASPP module is employed to extract and select multiscale features, focusing on complex water body boundary details. Additionally, a series of LGFF modules are inserted between the encoder and decoder to reduce the semantic gap between the encoder and decoder feature maps and the spatial information loss caused by the encoder’s downsampling process, improving the model’s ability to learn detailed information. Sentinel-1 SAR data from the Qinghai–Tibet Plateau region are selected, and the water extraction performance of the proposed LGFUNet model is compared with that of existing methods such as U-Net, Swin-UNet, and SCUNet++. The results show that the LGFUNet model achieves the best performance, respectively.

## 1. Introduction

Surface water resources are crucial water resources on Earth, supporting indispensable functions related to human production, daily life, and the material and energy cycles [1,2,3,4,5]. Satellite remote sensing technology has been widely applied in surface water detection because of its advantages of a fast monitoring speed, strong timeliness, and ability to obtain large-scale repetitive observations. Compared with optical remote sensing, synthetic aperture radar (SAR), as an active microwave system, can penetrate clouds, rain, and fog and is unaffected by lighting conditions, enabling all-weather, day–night target sensing. SAR systems can obtain information about objects by actively emitting radar wave signals and receiving backscattered echo signals from the Earth’s surface. When the radar signal emitted by a SAR system encounters a water surface, it typically exhibits specular reflection, with only a small portion of the signal returning to the receiving system. In contrast, rough surface objects return stronger backscattered signals via mechanisms such as diffuse reflection, secondary scattering, or volume scattering. Therefore, by utilizing the differences in backscatter values between water and non-water objects in SAR images, it is possible to distinguish water bodies effectively. This characteristic makes SAR an invaluable tool for water extraction, with enormous potential [6].

There are two main types of methods for extracting water from SAR images: traditional methods and deep learning methods [4]. Traditional methods primarily include thresholding [7,8,9], clustering analysis [10,11,12,13,14], Markov random field (MRF) [15], and machine learning approaches [16]. However, these methods are often influenced by the heterogeneity of the observation environment and the inherent speckle noise in SAR images [17], making it difficult to meet the current demand for rapid, large-scale surface water extraction. Machine learning methods, represented by support vector machines (SVMs) and random forests (RFs) [18,19], can learn the differences in the patterns of water and non-water bodies across multiple feature dimensions through training datasets, thus improving the water extraction accuracy to some extent. However, most of the features in these methods rely on manual design, and their feature representation capabilities are limited, making it difficult to ensure that they can fully capture the distinctions between water bodies and other land features. In contrast, deep learning techniques possess powerful feature learning capabilities and do not require manual feature design [20], providing a new approach for water extraction from SAR images.

Over the past few decades, deep learning has undergone rapid advancements and has been extensively applied in various SAR remote sensing tasks, such as image classification [21,22,23,24], change detection [25,26], and target recognition [27,28,29,30,31]. In 2015, Jonathan Long proposed fully convolutional networks (FCNs) [32], which replaced the fully connected layers of traditional CNNs with convolutional layers, marking the first application of deep learning in the field of semantic segmentation. Kang et al. used a FCN for flood monitoring and mapping with Gaofen-3 SAR imagery [33], but the FCN model failed to adequately consider the global spatial relationships among pixels [34], resulting in poor fine-scale segmentation. Ronneberger et al. enhanced the architecture of FCNs by increasing the number of decoders and incorporating skip connections to link decoder and encoder features, leading to the development of a U-Net network with an encoder–decoder structure [35]. Wang et al. compared the water extraction accuracies of three methods—the Otsu method, the object-oriented method, and U-Net—using Sentinel-1 SAR imagery and demonstrated the advantages of the U-Net model for water extraction [36]. However, owing to the similar backscattering characteristics of water bodies and land features, such as mountain shadows in SAR images [25], there can be confusion between the two during water extraction, especially in mountainous regions. Although the U-Net approach has improved water extraction compared with traditional methods, it still struggles to effectively address issues such as confusion between water bodies and shadows, and the accurate extraction of complex boundary details for continuous water bodies may be limited.

Owing to its robust generalization capabilities supported by the U-shaped encoder–decoder architecture, U-Net has emerged as one of the predominant networks for semantic segmentation and water body extraction from SAR images [5]. Researchers have proposed numerous enhanced network models that are based on the U-Net framework. For example, H. Song et al. proposed an improved U-Net network based on a hybrid attention mechanism (HA-UNet) for urban water extraction [37]. By conducting experiments with Sentinel-1A SAR images, they demonstrated that the proposed method significantly improved the accuracy of water body extraction in urban areas. Chuan Xu et al. developed a flood detection method for SAR images by integrating attention U-Net with a multiscale level set method and applied it for the dynamic monitoring of flood disasters in Jiangxi, Anhui, and Chongqing in 2020 [38]. However, this method still faces challenges in terms of water extraction in mountainous areas. Wang introduced dilated convolution and the spatial channel squeeze and excitation (SCSE) attention mechanism to U-Net [39], proposing a floodwater extraction network (FWENet) based on SAR images [40]. This model improved the extraction of small water bodies and water body boundaries, but the distinction between water bodies and shadows remained suboptimal. However, existing studies that utilize deep learning for SAR image-based water extraction rely primarily on convolutional kernels as the core for feature extraction. Convolution kernels are limited by their receptive fields [4], hindering their ability to capture global information effectively from SAR images. Challenges such as confusion between shadows and water bodies, as well as difficulties in extracting the detailed boundaries of continuous water bodies, persist.

To address the aforementioned issues, a novel end–end network framework called the local and global feature fusion UNet (LGFUNet) model is developed in this study and applied for water extraction in the complex terrain of the Qinghai–Tibet Plateau to demonstrate the advantages of the proposed method. The main contributions of this paper are as follows:

(1) A multiscale feature learning module (DECASPP) is proposed. Without increasing the number of model layers, DECASPP enables the model to learn important multiscale water body features, thereby enhancing the model’s ability to distinguish between shadows and water bodies.
(2) To address the issue of the misdetection of small water bodies, the local and global feature fusion (LGFF) module is introduced; it integrates the global and local features of water bodies, improving the model’s ability to extract detailed information about small water bodies.
(3) To address the challenge of extracting complex boundary details for continuous water bodies, the LGFUNet water extraction network model is established by combining the Swin-Transformer [41], DECASPP, and LGFF modules. This model comprehensively learns both the global and local features of water bodies at different scales, enhancing its ability to extract large-scale water bodies while effectively preserving the boundary details of spatially continuous and complex water bodies.

## 2. Methods

### 2.1. Overall Structure of the Proposed LGFUNet Model

**Figure 1:**
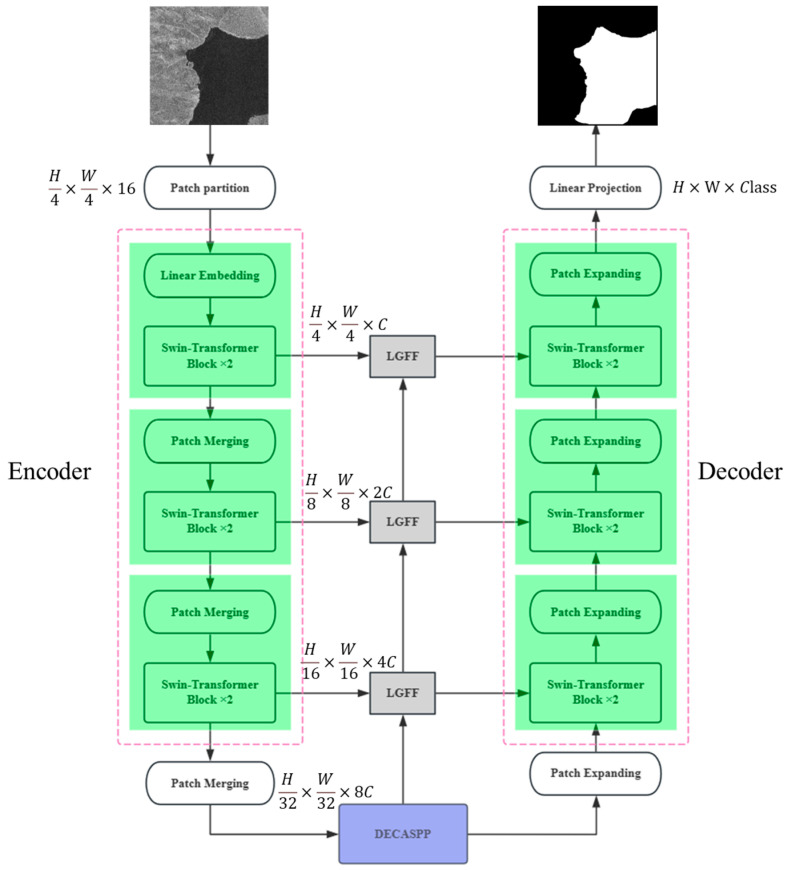
Overall structure of the proposed LGFUNet.

The overall architecture of the proposed LGFUNet model is shown in Figure 1. The model consists of three main modules: the encoder–decoder module, the DECASPP module, and the LGFF module. The LGFUNet model begins by transforming the input image into patch tokens of size H/4 × W/4 and dimension C through a patch partitioning layer and a linear embedding layer. These patch tokens are then passed through the Swin-Transformer block to learn the global information associated with continuous water bodies. The features are subsequently downsampled through the patch merging layer, and the Swin-Transformer block is utilized to learn global water body information at different scales, sequentially obtaining multiscale image features with dimensions *H*/4 × *W*/4 × *C*, *H*/8 × *W*/8 × 2*C*, *H*/16 × *W*/16 × 4*C* and *H*/32 × *W*/32 × 8*C*. At the end of the encoder stage, the features are passed to the DECASPP module, which further captures water body information at various scales without downsampling, providing valuable multiscale features for the subsequent encoder. During the decoder phase, the model employs the patch expanding layer to upsample the features, gradually restoring the feature size until it matches the input image dimensions. However, upsampling alone cannot fully recover the complete image features. To prevent information loss, the model incorporates a series of LGFF modules between the decoder and encoder. These modules autonomously filter detailed water body features while implementing skip connections for the corresponding layers in the decoder. Furthermore, the Swin-Transformer block is used to fuse the upsampled feature information with the salient water body features transmitted by the LGFF, thus integrating global and local information at different scales. Finally, the multiscale feature image generated by the decoder, which matches the input image size (*H* × *W*), undergoes linear projection to obtain pixel-level water body extraction results.

### 2.2. Swin-Transformer Block

**Figure 2:**
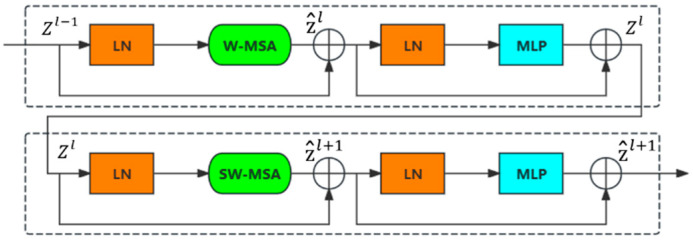
Swin-Transformer block.

As illustrated in Figure 2, the Swin-Transformer block consists of two consecutive structures, each comprising two LayerNorms (LNs), a multilayer perceptron (MLP) with GELU nonlinearity, a residual connection, and a multihead self-attention (MSA) module. The MSA modules in these two structures utilize window-based MSA (W-MSA) and shift window-based MSA (SW-MSA). Compared with convolutional architectures, the Swin-Transformer block enhances the model’s ability to capture long-range dependencies and learn the spatially continuous features of large-scale water bodies by implementing window-shifting operations through SW-MSA, which establishes connections across different self-attention windows.

### 2.3. DECASPP

**Figure 3:**
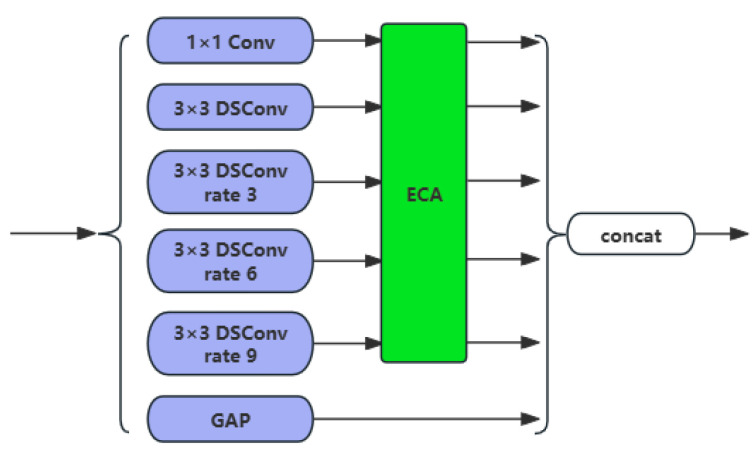
DECASPP structure.

Owing to the limitations of the Swin-Transformer block in extracting local image features, this paper integrates efficient channel attention (ECA) [42], depthwise separable convolution [43], and atrous spatial pyramid pooling [44] to construct the DECASPP module. Comprising six parallel branches, as illustrated in Figure 3, DECASPP includes four parallel attention pooling branches formed by depthwise separable convolution (DSConv) with different dilation rates, a 1 × 1 convolution mapping branch combined with ECA, and a global average pooling (GAP) branch. The input feature dimension of the module is 8C. GAP is responsible for downsampling the features to prevent overfitting in this layer. The four depthwise separable convolution branches with different dilation rates and the 1 × 1 convolution mapping branch are designed to capture contextual information for water bodies from various receptive fields. The depthwise separable convolution steps reduce the number of parameters while maintaining model performance, improving model efficiency. Additionally, the ECA mechanism is applied to filter multiscale features in the channel dimension, further enhancing the multiscale extraction effect. This helps provide rich multiscale features for the subsequent decoder and LGFF module. In the DECASPP module, the feature dimension and resolution remain unchanged.

ECA [42] can capture inter-channel dependencies without the need for dimensionality reduction or expansion. First, ECA performs global average pooling on the input features, and then it executes a size-k fast one-dimensional convolution based on each channel and its *k* neighboring channels to generate channel weights, thereby capturing the dependencies between channels. The value of *k* is adaptively determined through the channel dimension, as shown in the following formula:

(1) $$k=ѱ(C)={\left|\frac{\log_{2}\left(C\right)}{y}+\frac{b}{y}\right|}_{odd}$$

In the expression, |t|_odd_ denotes the nearest odd number to t, with b set to 1 and y set to 2; C represents the number of input channels.

For convolutional operations with predetermined input and output feature map dimensions as well as kernel sizes, the parameter counts for standard convolution and depthwise separable convolution are expressed as follows:

(2) $$S_{conv}=M\times {(D}_{K}\times D_{K}\times N)$$

(3) $$S_{DSC}={M\times (D}_{K}\times D_{K}+N)$$

where *M* denotes the number of input feature channels, N represents the number of output feature channels, and the convolution kernel size is *D_K_* × *D_K_*. *S_conv_* and *S_DSC_* correspond to the parameter counts of standard convolution and depthwise separable convolution, respectively, under the assumption of no bias terms. The effectiveness of depthwise separable convolution in reducing parameters becomes more pronounced as the number of output feature channels increases.

### 2.4. LGFF

**Figure 4:**
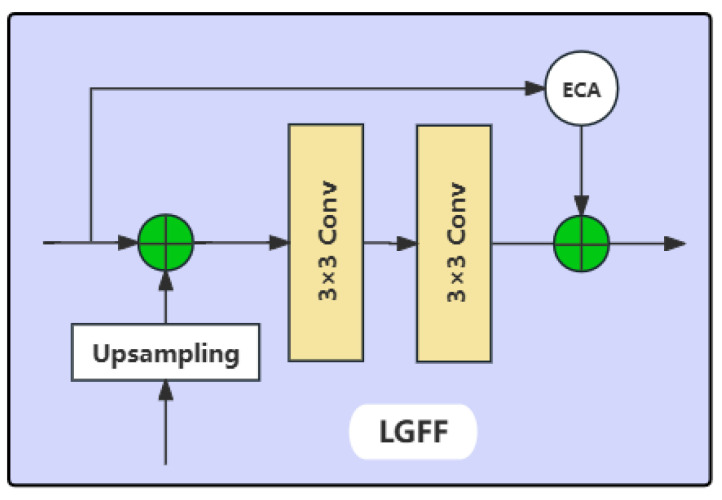
The LGFF structure.

The conventional skip connections in the U-Net architecture may inadequately bridge the semantic gap between the features of the encoder and decoder. To address this issue, we integrate a series of LGFF modules between the encoder and decoder, as illustrated in Figure 4. The LGFF module employs convolutional operations to extract local features from the water body information conveyed by the encoder and the DECASPP module, thereby compensating for the limitations of the Swin-Transformer structure in capturing detailed water body features and providing additional information to the decoder. The ECA mechanism is used to selectively filter the global features extracted by the encoder, and the refined global features are then combined with the local features extracted by the LGFF. This combined feature set is transmitted to the decoder to diminish the semantic disparity between the encoder and decoder, ameliorate the loss of spatial information during the encoder’s patch merging and downsampling processes, and enhance the model’s ability to learn intricate details of water bodies.

## 3. Experiment

### 3.1. Dataset

**Figure 5:**
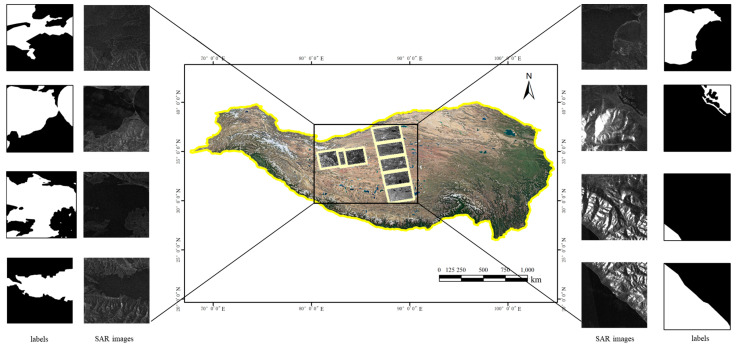
Examples from the SAR sample dataset.

**Figure 6:**
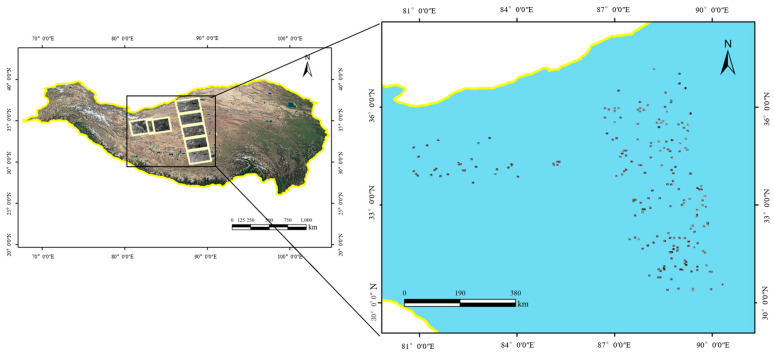
Examples from the test dataset.

In this study, the Qinghai–Tibet Plateau is the experimental area used to verify the advantages of the proposed algorithm. The plateau is dotted with numerous water bodies of varying sizes, including China’s largest inland saltwater lake, Qinghai Lake, as well as countless smaller lakes (with areas of less than 1 km^2^). Additionally, the Qinghai–Tibet Plateau is characterized by dense mountains and complex terrain. Water body extraction research in this region requires not only the effective differentiation of mountain shadows and water bodies but also the identification of small water bodies and the complex boundaries of spatially continuous water bodies. Seven Sentinel-1 SAR images from the Qinghai–Tibet Plateau region are used to construct a water body dataset, with the basic information for the data sources presented in Table 1. The original data underwent preprocessing steps such as radiometric correction and geocoding, resulting in orthorectified images that each pixel represents a ground extent of 14 × 14 meters meters. These images, combined with a manual visual interpretation of Google optical imagery, were used to delineate ground truth water body labels. For training and validation purposes, the water body labels and the seven SAR images were divided into 3083 sample patches of size 512 × 512. Among these, 2573 image samples were randomly selected as the training dataset, 201 image samples were selected as the test dataset, and the remaining samples were selected as the validation dataset. The study area and dataset examples are illustrated in Figure 5. In the labels, the black and white regions denote non-water and water bodies, respectively. The location distribution of the test dataset is shown in Figure 6.

The network proposed in this study was constructed and developed via PyTorch 1.12.1, CUDA 11.3, and Python 3.10. It was trained on an Nvidia Quadro Rtx 5000 with 16 GB of GPU memory. Given the GPU memory constraints, the batch size was set to 4. The Adam optimization algorithm was employed to update the model gradients, with an initial learning rate of 0.001.

### 3.2. Comparison of Overall Performance Among Different Models

To facilitate a quantitative analysis of model performance, this study employs a confusion matrix derived from the comparison between predicted images and ground truth labels, as illustrated in Table 2. The four fundamental metrics within the confusion matrix are defined as follows:

True Positive (TP): The number of water pixels correctly classified as water.

True Negative(TN): The number of non-water pixels correctly classified as non-water.

False Positive (FP): The number of non-water pixels erroneously classified as water.

False Negative(FN): The number of water pixels erroneously classified as non-water.

To validate the effectiveness of the proposed model, we conducted comparative experiments between the LGFUNet model and several state-of-the-art networks, namely, U-Net, Swin-UNet [45], and SCUNet++ [46]. To comprehensively evaluate the performance of the LGFUNet model in water body extraction, we employed the following indicators: overall accuracy (OA), precision, recall, F1-score, intersection over union (IoU), and kappa [37]. OA represents the ratio of correctly classified pixels to the total number of pixels. Precision is the ratio of correctly predicted water pixels to all predicted water pixels. Recall is the ratio of correctly predicted water pixels to the actual number of water pixels. The F1-score balances precision and recall. The IoU is the ratio of the intersection to the union of the predicted water regions and the true water regions. Kappa is used to assess the degree of agreement between the water extraction results and the actual ground reference data. The definitions of these indicators are shown in Equations (4)–(9) as follows:

(4) $$OA=\frac{TP+TN}{TP+FN+FP+TN}$$

(5) $$Precision=\frac{TP}{TP+FP}$$

(6) $$Recall=\frac{TP}{TP+FN}$$

(7) $$F1-Scores=\frac{2TP}{2TP+FN+FP}$$

(8) $$IoU=\frac{TP}{TP+FP+FN}$$

(9) $$Kappa=\frac{OA-Pe}{1-Pe},\;Pe=\frac{\left(TP+FN\right)\left(TP+FP\right)+(FP+TN)(FN+TN)}{{\left(TP+TN+FP+FN\right)}^{2}}$$

The quantitative accuracy evaluation results for the LGFUNet model compared with the other models for the test dataset are presented in Table 3. Compared with the other methods, the proposed LGFUNet model demonstrates superior water extraction accuracy overall, with significant improvements across all the indicators. Specifically, the LGFUNet model achieves an OA of 99.31%, a precision of 96.31%, a recall of 92.50%, an F1-score of 94.04%, an IoU of 89.48%, and a kappa coefficient of 93.40%. Compared with the Swin-UNet model, the LGFUNet model yields improvements of 0.92%, 1.07%, 1.60%, 1.52%, 2.45%, and 1.78% across the respective indicators. These results indicate that the LGFUNet model can effectively identify and extract surface water bodies on the Qinghai–Tibet Plateau.

To provide an accurate and intuitive comparison of the results of these methods, we selected several typical areas within the study area for model performance comparisons. This comparison aimed to verify the advantages of the method proposed in this paper in terms of small lake water body extraction, the distinction between shadows and water bodies, and the preservation of complex boundary details for continuous water bodies.

### 3.3. Performance Comparison for the Identification of Small Lakes

**Figure 7:**
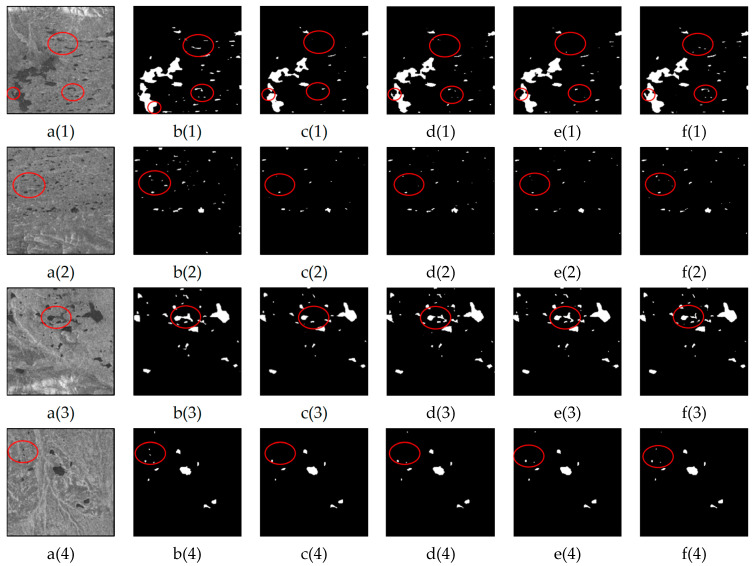
Performance comparison of different models for small lake water extraction. (**a(1)**–**a(4)**) represent the SAR images; (**b(1)**–**b(4)**) depict the corresponding ground truth features for the SAR images; (**c(1)**–**c(4)**), (**d(1)**–**d(4)**), (**e(1)**–**e(4)**), and (**f(1)**–**f(4)**) display the extraction results of U-Net, Swin-UNet, SCUNet++, and LGFUNet, respectively. In the extraction results, the black and white regions denote non-water and water bodies, respectively. The red circles highlight key areas where prediction errors occur.

The extraction of small lakes is crucial for water body extraction tasks. In this study, we compared the LGFUNet model with three other models in small water body areas, and the results are illustrated in Figure 7. The visualization demonstrates that the proposed LGFUNet model excels in identifying and extracting small lake water bodies from SAR images, with the overall performance being superior to that of the other models. While U-Net achieves high precision, it tends to be overly conservative in extracting small water bodies, failing to capture all water bodies effectively, with significant omissions particularly evident in Figure 7(c(1),c(2),c(4)). As shown in Figure 7(d(1),d(2)), Swin-UNet displays a comparatively worse ability to extract local features, resulting in lower precision for small water bodies. SCUNet++ performs slightly better than Swin-UNet in extracting small water bodies but still falls short of the LGFUNet model in terms of its overall performance. The visual results highlight that the LGFUNet model significantly improves the extraction of small water bodies compared with the other models, although some omissions are observed at the blurred boundaries of small water bodies in Figure 7(f(3)). A quantitative evaluation of the prediction results on the basis of the images in Figure 7 is presented in Table 4. Compared with the other models, the LGFUNet model performs best in small water body extraction. Specifically, compared with the Swin-UNet model, the LGFUNet model achieves improvements of 0.12%, 2.19%, 1.82%, 2.30%, 3.16%, and 2.37% for OA, recall, F1-score, IoU, and kappa, respectively.

### 3.4. Performance Comparison for Water Body Identification Within Shadowed Areas

**Figure 8:**
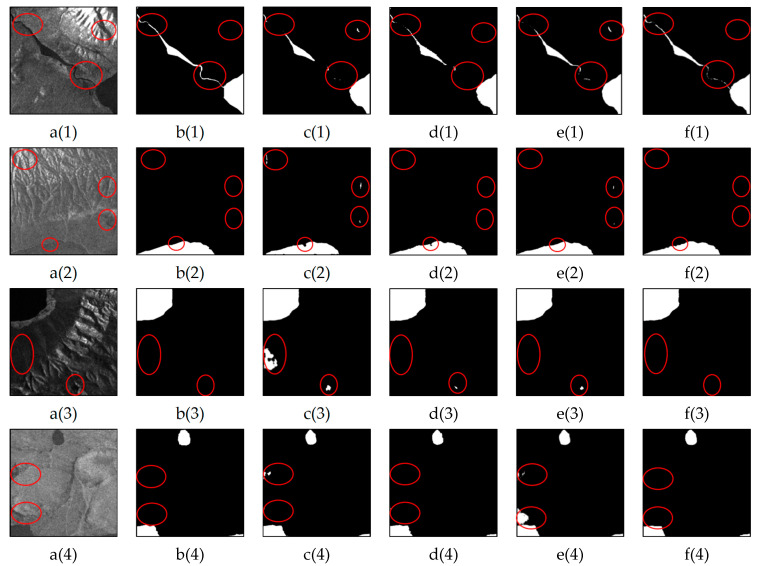
Performance comparison of different models for water extraction in shadow areas. (**a(1)**–**a(4)**) represent the SAR images; (**b(1)**–**b(4)**) depict the corresponding ground truth features for the SAR images; (**c(1)**–**c(4)**), (**d(1)**–**d(4)**), (**e(1)**–**e(4)**), and (**f(1)**–**f(4)**) display the extraction results of U-Net, Swin-UNet, SCUNet++, and LGFUNet, respectively. In the extraction results, the black and white regions denote non-water and water bodies, respectively. The red circles highlight key areas where prediction errors occur.

Shadows are among the most significant challenges when extracting water bodies from SAR images. In SAR images, shadows exhibit backscattering characteristics similar to those of water bodies (both appear as dark areas), leading to confusion between the two during water extraction, particularly in mountainous regions. Figure 8 presents the water extraction results of the LGFUNet model compared with those of the other models in shadow areas. The U-Net model demonstrates a poor ability to distinguish between water bodies and shadows, as is particularly evident in Figure 8(c(3),c(4)). In Figure 8(e(1),e(4)), the SCUNet++ model fails to differentiate between water bodies and shadows, resulting in significant misclassification. The visualization results show that, compared with the other models, the LGFUNet model performs best in identifying water bodies in shadow areas, with a noticeable reduction in misclassifying shadows as water. However, some omissions of small water bodies in shadow areas are still observed in Figure 8(f(1)). A quantitative evaluation of the prediction results based on the images in Figure 8 is presented in Table 5, and the findings indicate that the LGFUNet model outperforms the other models across all indicators.

### 3.5. Performance Comparison for Identifying Complex Spatially Continuous Water Bodies

**Figure 9:**
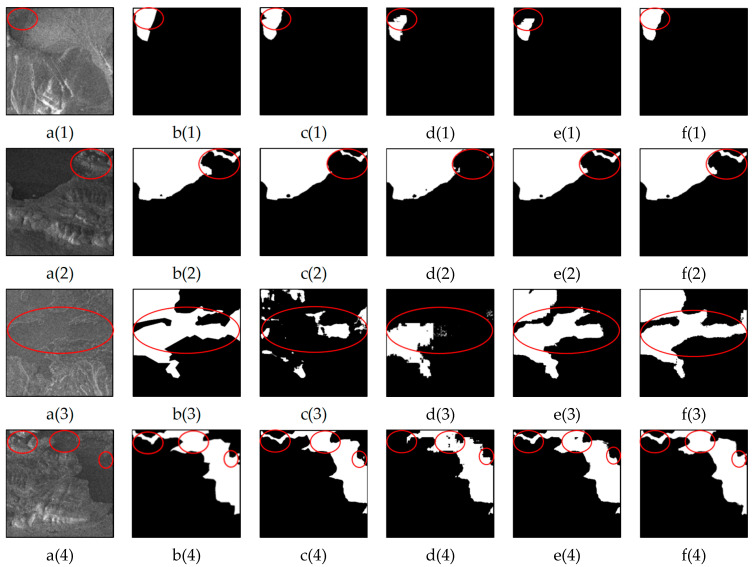
Performance comparison of different models in extracting the detailed boundaries of spatially continuous water bodies. (**a(1)**–**a(4)**) represent the SAR images; (**b(1)**–**b(4)**) depict the corresponding ground truth features for the SAR images; (**c(1)**–**c(4)**), (**d(1)**–**d(4)**), (**e(1)**–**e(4)**), and (**f(1)**–**f(4)**) display the extraction results of U-Net, Swin-UNet, SCUNet++, and LGFUNet, respectively. In the extraction results, the black and white regions denote non-water and water bodies, respectively. The red circles highlight key areas where prediction errors occur.

SAR acquires information about ground objects by capturing reflected radar wave signals, which results in similar backscattering characteristics between water bodies and surrounding low-reflectance objects in SAR images. This makes it challenging to distinguish spatially continuous water bodies with complex boundaries from transition areas that exhibit similar backscattering characteristics. Accurately extracting the detailed boundaries of spatially continuous water bodies that resemble surrounding ground features is a significant challenge. The extraction results of the LGFUNet model and other models for this type of water body are illustrated in Figure 9.

In Figure 9, for the water body region in Figure 9(a(3)), with complex spatial continuity and features that resemble those of surrounding ground objects, both U-Net and Swin-UNet struggle to distinguish the water body from other ground objects, demonstrating poor model performance. In Figure 9(a(1)), Swin-UNet and SCUNet++ exhibit inadequate performance in extracting water bodies from areas with similar surrounding ground features. Additionally, in Figure 9(d(2),d(4)), Swin-UNet performs poorly in extracting the detailed boundaries of spatially continuous water bodies. In contrast, the LGFUNet model demonstrates superior performance across Figure 9(f(1)–(4)). Notably, in Figure 9(f(3)), the LGFUNet model yields a significant improvement over the other models, although it still fails to fully extract small river branches with complex spatial continuity for water body boundaries. A quantitative evaluation of the prediction results based on the images in Figure 9 is presented in Table 6. Compared with the Swin-UNet model, the proposed LGFUNet model achieves improvements of 6.30%, 27.73%, 18.73%, 26.21%, and 21.72% in OA, recall, F1-score, IoU, and kappa, respectively.

## 4. Discussion

The proposed LGFUNet model in this study employs the Swin-Transformer as a feature extractor, incorporating several key innovations. First, a DECASPP module is constructed, which combines atrous spatial pyramid pooling, ECA, and depthwise separable convolution to enhance multiscale feature extraction and obtain valuable multiscale information. Second, a series of LGFF modules are introduced between the encoder and decoder. These modules integrate global features from the encoder, multiscale features extracted by DECASPP, and local features extracted by the LGFF modules, which are then passed to the decoder. This integration reduces the semantic gap between the encoder and decoder feature maps and mitigates spatial information loss caused by patch merging and downsampling, thereby improving the model’s ability to learn detailed information. As demonstrated by the visualization results and quantitative evaluations in Chapter 3, the LGFUNet model exhibits superior performance in extracting water bodies in complex regions, including small water bodies, spatially continuous water bodies, and shadow areas. The number of parameters for each model and the training time per epoch are shown in Table 7. The LGFUNet model has enhanced learning capabilities for water feature characteristics in SAR images, resulting in a slight increase in the number of parameters; however, it has a certain advantage in training time. Moreover, the LGFUNet model demonstrates the best performance across various metrics on the test dataset.

**Figure 10:**
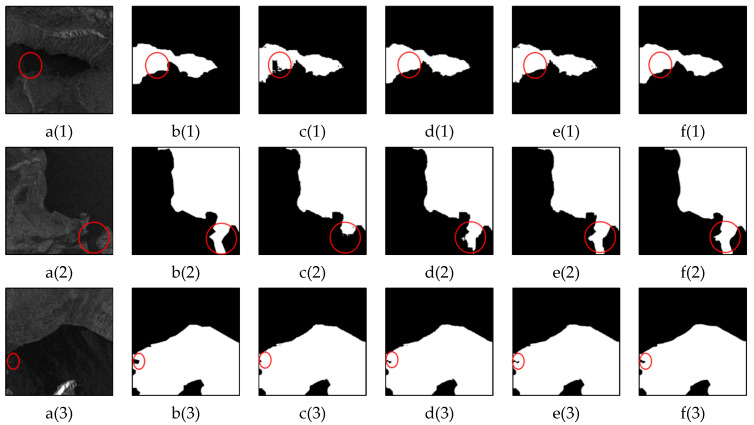
Performance comparison of different models in the ablation experiment. (**a(1)**–**a(3)**) represent the SAR images; **(b(1)**–**b(3)**) depict the corresponding ground truth features for the SAR images; (**c(1)**–**c(3)**), (**d(1)**–**d(3)**), (**e(1)**–**e(3)**), and (**f(1)**–**f(3)**) display the extraction results of Swin-UNet, Swin-UNet+DECASPP, Swin-UNet+LGFF, and LGFUNet, respectively. In the extraction results, the black and white regions denote non-water and water bodies, respectively. The red circles highlight key areas where prediction errors occur.

To further validate the effectiveness of the proposed improvements in the LGFUNet model, ablation experiments were conducted to assess the impact of the DECASPP and LGFF modules. The results of the ablation experiments are presented in Table 8, and the visualization results are shown in Figure 10. In Swin-UNet+DECASPP, the DECASPP module alone is employed to enhance multiscale feature extraction, effectively improving the accuracy of water body extraction. However, the improvements in the other metrics are relatively modest. In Figure 10(d(1),d(2)), this module significantly enhances Swin-UNet’s ability to extract detailed water body boundaries. In Swin-UNet+LGFF, only the LGFF module is used to reduce the semantic gap between the encoder and decoder feature maps, improving Swin-UNet’s ability to extract local features. All the evaluation metrics for the prediction results show improvement. In Figure 10(e(1),e(3)), the LGFF module enhances Swin-UNet’s ability to extract small water bodies, although the overall model performance remains inferior to that of the LGFUNet model. The experiments demonstrate that the LGFF module can effectively enhance the model’s water extraction performance. While the DECASPP module alone does not significantly improve model performance, it provides the LGFF module with valuable multiscale information. When the DECASPP module is combined with the LGFF module, the model achieves optimal performance.

Siling Co (located in Nagqu City, Tibet Autonomous Region) is one of China’s largest inland saltwater lakes. Research by Lei Yanbin et al. [47]. has revealed that Siling Co has become one of the most rapidly expanding lakes on the Qinghai–Tibet Plateau over the past two decades. Its rapid expansion has led to the inundation of surrounding grasslands and road damage, significantly impacting the local environment, wildlife, and human livelihoods. Therefore, this study selected Siling Co to validate the model’s generalization capability and explored the correlation between lake area and meteorological data based on the monitoring results.

**Figure 11:**
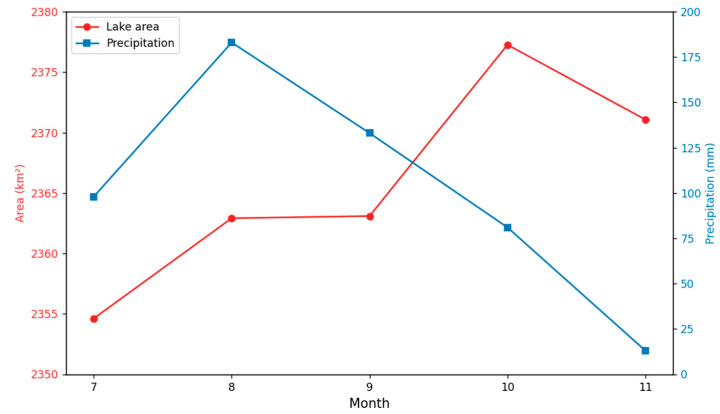
The monthly variations in Siling Co’s surface area and average precipitation from July to November 2018.

This research utilizes Sentinel-1A images acquired at the beginning of each month from July to November 2018 for the Siling Co region. Combined with LGFUnet extraction, we investigated the short-term fluctuations in the lake’s monthly water extent. The monthly variations in the water surface area of Siling Co Lake and the corresponding average precipitation from July to November 2018 are presented in Figure 11.

The Siling Co region experiences its rainy season from July to September, characterized by substantial monthly precipitation. During this period, the surface area of Siling Co Lake exhibits a steady expansion, reaching its maximum extent at the end of the rainy season (early October). Following the conclusion of the rainy season, the lake gradually enters its freezing period. During freezing, precipitation and evaporation on the Qinghai–Tibet Plateau are both minimal, resulting in relatively stable Siling Co lake areas. Consequently, this study employs July Sentinel-1A data from 2017 to 2024 to monitor Siling Co’s interannual variations.

**Figure 12:**
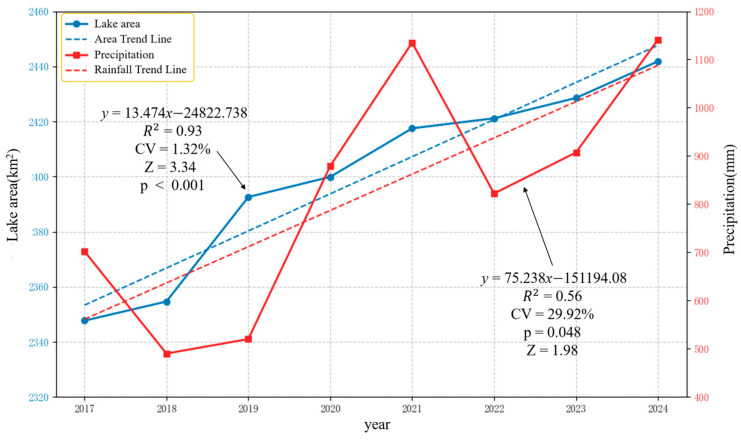
Line chart of the area of Siling Co Lake and precipitation from 2017 to 2024.

The interannual variations in the Siling Co Lake area and annual precipitation from 2017 to 2024 are presented in Figure 12. The lake area exhibited a minimum value of 2347.7 km^2^ in 2017 and reached its maximum of 2441.82 km^2^ in 2024, demonstrating a consistent expansion trend. Over the eight-year period, the lake area increased by 94.12 km^2^ in total, with an average annual growth rate of 0.5%. Precipitation showed an overall increasing trend with considerable interannual variability (coefficient of variation, CV = 29.92%), indicating significant year-to-year differences in rainfall.

To quantitatively assess the relationship between lake area and climatic factors, we performed Pearson correlation analysis. The results revealed a statistically significant positive correlation (r = 0.76, *p* = 0.029) between lake area and precipitation, suggesting a strong association (*p* < 0.05). For instance, the annual precipitation in 2021 (from July 2020 to July 2021) reached 1135 mm, corresponding to an area increase of 17.63 km^2^, while the lower precipitation in 2018 (490 mm) resulted in a smaller expansion of 6.93 km^2^. Precipitation during the previous year’s rainy season (July–September) is stored through surface runoff and winter ice formation, then gradually released during spring snowmelt, ultimately influencing the lake area measured in July.

## 5. Conclusions

In this study, a water extraction method based on SAR imagery, the LGFUNet model, is proposed. Sentinel-1A SAR images are used as the basis for extraction, and the model achieves promising extraction results in the study area on the Qinghai–Tibet Plateau. In the LGFUNet model, the Swin-Transformer module is employed to replace convolutional kernels for feature extraction, enhancing the learning of global features and improving the model’s ability to capture the spatial relationships associated with large, continuous water bodies. Within the DECASPP module, ECA and atrous spatial pyramid pooling are utilized to filter and refine multiscale features, thereby improving multiscale extraction performance. Additionally, a series of LGFF modules are introduced between the encoder and decoder. These modules integrate global information from the encoder, multiscale feature information from the DECASPP module, and local information extracted by the LGFF modules, which are then passed to the decoder. This integration reduces the semantic gap between the encoder and decoder feature maps, compensates for Swin-Transformer’s limitations in local feature extraction, mitigates spatial information loss during downsampling, and enhances the model’s ability to extract small water bodies. Both quantitative evaluation results and visualization results comparing the LGFUNet model with other models demonstrate that the LGFUNet model can accurately and effectively extract surface water resources on the Qinghai–Tibet Plateau, showing significant potential for water body extraction applications in this region.

In comparative experiments involving three challenging tasks—water extraction in shadowed areas, small lake extraction, and complex boundary extraction for continuous water bodies—the proposed LGFUNet model outperforms other end-to-end models, demonstrating superior performance. However, some limitations remain, such as occasional omissions of small water bodies with blurred boundaries and fine river branches with spatially continuous and complex boundaries. In future work, we aim to further refine the LGFUNet model, including but not limited to the following aspects: exploring more combinations of the Swin-Transformer and CNNs based on the LGFUNet architecture to optimize the model structure and enhance performance; expanding the dataset to improve the model’s generalization capabilities; and addressing the current limitations to achieve more robust and accurate water body extraction.

## Figures and Tables

**Table 1:** Basic information for the data sources.

Sentinel-1A	Parameter
Product format	SLC
Product level	Level-1
Radar wave frequency	5.4 GHz
Beam mode	Interferometric wide swath
Polarization	VV
Resolution	13.9 m × 2.3 m
Image size	20,148 × 15,774

**Table 2:** The confusion matrix.

		Prediction	
		Water	Background
**Ground Truth**	Water	True Positive (TP)	False Negative (FN)
	Background	False Positive (FP)	True Negative (TN)

**Table 3:** Metrics of the models for the test dataset.

	U-Net	Swin-UNet	SCUNet++	LGFUNet
OA(%)	96.87	98.39	98.98	**99.31**
Precision (%)	93.27	95.24	95.60	**96.31**
Recall (%)	86.48	90.89	90.90	**92.50**
F1-score (%)	87.43	92.52	92.42	**94.04**
IoU (%)	80.95	98.39	98.98	**99.31**
Kappa (%)	85.32	95.24	95.60	**96.31**

**Table 4:** Model metrics for the images in Figure 7.

	U-Net	Swin-UNet	SCUNet++	LGFUNet
OA(%)	99.03	99.13	99.21	**99.25**
Precision (%)	97.55	92.06	94.97	94.25
Recall (%)	69.19	77.46	76.12	**79.28**
F1-score (%)	79.51	83.32	83.10	**85.62**
IoU (%)	67.75	72.41	72.73	**75.57**
Kappa (%)	79.02	82.87	82.70	**85.24**

**Table 5:** Model metrics for the images in Figure 8.

	U-Net	Swin-UNet	SCUNet++	LGFUNet
OA(%)	98.52	99.38	98.92	**99.62**
Precision (%)	93.59	98.60	92.32	**99.17**
Recall (%)	91.11	93.33	93.07	**94.06**
F1-score (%)	92.10	95.83	92.30	**96.51**
IoU (%)	85.38	92.12	86.38	**93.36**
Kappa (%)	88.55	94.61	89.53	**96.31**

**Table 6:** Model metrics for the images in Figure 9.

	U-Net	Swin-UNet	SCUNet++	LGFUNet
OA(%)	91.80	91.92	96.89	**98.31**
Precision (%)	98.43	97.42	97.98	97.47
Recall (%)	75.35	67.32	79.84	**95.05**
F1-score (%)	81.19	77.47	86.74	**96.20**
IoU (%)	74.32	66.53	78.86	**92.74**
Kappa (%)	77.83	73.26	84.66	**94.98**

**Table 7:** Comparison of the parameter amounts and training times of various models.

Models	Number of Trainable Parameters	Training Time per Epoch (s)
U-net	3.1 × 107	667
Swin-Unet	4.2 × 107	403
SCUNet++	6.3 × 107	629
LGFUNet	7.2 × 107	460

**Table 8:** Metrics for the ablation experiment.

	Swin-Unet	Swin-Unet+DECASPP	Swin-Unet+LGFF	LGFUnet
OA(%)	98.39	99.16	98.97	**99.31**
Precision (%)	95.24	94.98	95.17	**96.31**
Recall (%)	90.89	91.94	91.31	**92.50**
F1-score (%)	92.52	93.06	92.78	**94.04**
IoU (%)	87.03	87.75	87.35	**89.48**
Kappa (%)	91.62	92.32	91.92	**93.40**

## Data Availability

The data that support the findings of this study are available from the corresponding author upon reasonable request.
